# Oral Hygiene Behaviors, Periodontal Awareness, and Self-Reported Periodontal Symptoms Among Adults: A Cross-Sectional Survey Study

**DOI:** 10.3390/healthcare14111570

**Published:** 2026-06-03

**Authors:** Mehmet Murat Taskan, Ozkan Karatas

**Affiliations:** Department of Periodontology, Faculty of Dentistry, Tokat Gaziosmanpasa University, Tokat 60250, Turkey; dtokaratas@hotmail.com

**Keywords:** periodontitis, oral hygiene, health knowledge, attitudes, surveys and questionnaires, self-report

## Abstract

**Highlights:**

**What are the main findings?**

- Higher periodontal awareness was associated with interdental cleaning and routine preventive dental attendance.
- Multivariable analysis identified complaint-based dental attendance, current smoking, and absence of interdental cleaning as independent correlates of reporting at least one periodontal symptom.
- Questionnaire-based screening may help identify preventable behavior profiles, but it cannot replace clinical periodontal diagnosis.

**Abstract:**

Objectives: The objective was to investigate associations among oral hygiene behaviors, periodontal awareness, and self-reported periodontal symptoms among adults using a cross-sectional questionnaire and an internally consistent awareness scoring system. Materials and Methods: This cross-sectional questionnaire study was conducted between October 2025 and February 2026 among adults aged 18–65 years. Of 412 returned questionnaires, 386 complete and eligible forms were analyzed. The 42-item questionnaire assessed sociodemographic variables, oral-hygiene behavior, dental attendance, previous periodontal care, awareness of periodontal signs, and self-reported symptoms during the previous six months. Awareness was scored from 0 to 20. Descriptive statistics, chi-square tests, *t* tests, one-way ANOVA with Tukey post hoc tests, and multivariable logistic regression were used. Results: Mean age was 31.8 ± 9.6 years, and 58.0% of participants were women. Twice-daily toothbrushing was reported by 56.0%, and interdental cleaning by 38.6%. Mean awareness score was 12.7 ± 3.8. Awareness was higher among participants with university/postgraduate education, regular dental attendance, and interdental cleaning habits (all *p* < 0.001). Gingival bleeding was the most frequently reported symptom (47.4%). In the adjusted analysis, complaint-based dental attendance (OR = 2.43; 95% CI: 1.46–4.03; *p* = 0.001), current smoking (OR = 1.96; 95% CI: 1.17–3.29; *p* = 0.011), and no interdental cleaning (OR = 2.08; 95% CI: 1.31–3.30; *p* = 0.002) were independently associated with reporting at least one periodontal symptom. Conclusions: Within the limits of this observational survey, preventive attendance and interdental cleaning were associated with higher awareness and lower self-reported symptom burden.

## 1. Introduction

Periodontal diseases are chronic inflammatory disorders of the tooth-supporting tissues and remain among the most common oral conditions affecting adults worldwide [1,2,3,4]. Contemporary classification and treatment frameworks emphasize that periodontal health, gingivitis, and periodontitis should be interpreted across biologically continuous states influenced by biofilm accumulation, host susceptibility, behavioral exposures, and preventive care [2,3,4,5,6,7].

Daily plaque control remains central to periodontal prevention. Although the historical experimental gingivitis literature established the biologic reversibility of plaque-induced gingival inflammation [8], current prevention guidelines place this concept within broader behavioral and supportive-care strategies, including individualized oral-hygiene instruction, interdental cleaning and regular professional maintenance [5,6,7,9,10,11], and smoking counseling [12,13,14,15].

Public awareness of periodontal disease remains incomplete. Many adults recognize caries more readily than early periodontal warning signs such as bleeding, swelling, recession, tooth mobility, or disease progression with little pain [16,17,18,19,20,21,22,23]. Low awareness may delay professional consultation and may normalize chronic symptoms, particularly when signs are intermittent or not painful.

Self-reported periodontal measures cannot replace full-mouth periodontal examination, but they are useful for surveillance, risk stratification, and health-education planning when clinical examinations are impractical [16,17,18,19,21,22,23]. Recent validation studies also show that self-reported items perform better when interpreted in combination with behavioral and demographic variables rather than as isolated diagnostic indicators [18,21,22].

Although studies have separately examined oral-hygiene habits, dental attendance, and self-reported periodontal disease, fewer questionnaire-based studies have integrated awareness scoring, preventive behavior, and symptom burden within a single analytical framework. This gap is important for preventive periodontology because knowledge may not automatically translate into sustained interdental cleaning or routine dental attendance. Previous studies on oral-health beliefs, dental attendance, social determinants, global periodontal burden, risk assessment, and professional maintenance provide a broader public-health context for these associations [24,25,26,27,28,29,30,31,32,33].

Therefore, the primary aim of this study was to determine whether oral-hygiene behaviors and preventive dental attendance were associated with higher periodontal awareness scores in adults. The secondary aim was to identify behavioral and demographic factors associated with self-reported periodontal symptom burden. Because of the cross-sectional design, all findings were interpreted as associations rather than causal relationships.

## 2. Materials and Methods

### 2.1. Study Design, Setting, and Period

This study was conducted as a cross-sectional questionnaire survey and was conceptually aligned with STROBE recommendations for observational studies [34]. Data were collected between 20 October 2025 and 20 February 2026 in the Tokat Gaziosmanpasa University Faculty of Dentistry, Department of Periodontology outpatient setting and affiliated community environment. Written informed consent was obtained from all participants. The protocol was approved by the Medical Ethics Committee of Tokat Gaziosmanpasa University (approval number: 2025/KAEK/12-10; approval date: 12 October 2025).

### 2.2. Participants, Recruitment, and Eligibility Criteria

The target population consisted of adults aged 18 to 65 years who were capable of reading and completing a self-administered questionnaire. Consecutive convenience sampling was used during the defined data-collection period. This non-probability recruitment strategy was acknowledged as a potential source of selection bias because individuals connected to dental services may differ from the general population in awareness and care-seeking behavior.

Inclusion criteria were: age between 18 and 65 years, ability to understand the questionnaire language, presence of at least one natural tooth, and voluntary written consent. Exclusion criteria were: complete edentulism, inability to complete the questionnaire independently, refusal to provide consent, ongoing acute dental emergency preventing questionnaire completion, and questionnaires with more than 10% missing or inconsistent responses.

### 2.3. Sample Size Rationale

The sample size was estimated for the primary comparison of awareness scores across oral-hygiene behavior groups. Assuming a medium effect size (f = 0.25), alpha = 0.05, and power = 0.80, at least 252 participants were required for one-way ANOVA with three exposure categories. To allow for incomplete responses, subgroup analysis, and multivariable logistic regression, planned enrollment was increased by more than 40%. A total of 412 forms were collected; 26 were excluded because of incomplete or inconsistent responses, leaving 386 questionnaires for analysis.

### 2.4. Questionnaire Development, Structure, and Response Format

The questionnaire was developed after a focused literature review on oral-hygiene behavior, self-reported periodontal symptoms, periodontal awareness, and self-report periodontal screening instruments [16,17,18,19,20,21,22,23,35,36]. The final instrument contained 42 items across four domains: 7 sociodemographic and background items, 12 oral-hygiene behavior and dental attendance items, 10 periodontal awareness items, and 13 symptom/treatment-history items. Most behavior and symptom items were categorical yes/no or multiple-choice questions. Awareness items used true/false/“I do not know” responses to reduce guessing and to distinguish lack of knowledge from incorrect knowledge.

Content relevance was reviewed by a periodontist (M.M.T.). A pilot administration was performed in 20 adults to assess clarity, completion time, and item redundancy. Minor wording revisions were made after pilot feedback. The questionnaire was not intended to establish a clinical diagnosis of periodontitis; rather, it was designed to assess awareness, behavior, and symptom reporting for epidemiologic and preventive-education purposes. This clarification was added to avoid overinterpretation of self-reported symptom items. The questionnaire structure and scoring details are provided in Appendix A.

### 2.5. Definition of Symptom Items and Participant Comprehension

Before symptom questions, participants were given brief lay definitions. Gingival bleeding was defined as bleeding during brushing, interdental cleaning, or spontaneous bleeding noticed in the mouth. Gingival swelling was defined as visible or felt enlargement/tenderness of the gums. Dentin hypersensitivity was described as short, sharp sensitivity to cold, sweet, or brushing stimuli. Persistent halitosis was described as repeated unpleasant breath noticed by the participant or others. Gingival recession was described as gums appearing to move downward/upward with teeth looking longer. Food impaction was defined as food frequently getting stuck between teeth. Tooth mobility was described as a tooth feeling loose during chewing or touch. These definitions were provided to improve comprehension and to reduce misclassification.

### 2.6. Awareness Score Construction and Reliability

Periodontal awareness was measured with 10 statements addressing plaque-induced gingival inflammation, the abnormality of gingival bleeding, interdental cleaning, smoking-related periodontal risk, gingival recession, tooth mobility, professional maintenance, painless progression, halitosis, and plaque control in the absence of pain. Each correct response received 2 points, whereas incorrect and “I do not know” responses received 0 points. The total score ranged from 0 to 20. For descriptive interpretation, awareness was categorized a priori as low (0–8), moderate (9–14), and high (15–20). These pragmatic bands were used only to summarize the score distribution and were not treated as externally validated diagnostic thresholds; similar score-banding approaches are common in health-knowledge questionnaire studies [20,23]. Internal consistency of the 10 awareness items was good (Cronbach’s alpha = 0.81).

### 2.7. Outcome Measures

The primary outcome was the continuous periodontal awareness score. The secondary outcome was self-reported periodontal symptom burden during the previous six months. Symptom items were selected because they are commonly used in periodontal self-report and surveillance studies and because they reflect symptoms that may motivate care-seeking behavior [16,17,18,19,21,22]. However, some items, including halitosis, dentin hypersensitivity, and food impaction, are not specific markers of periodontitis. Therefore, the regression outcome was described as self-reported periodontal symptom burden rather than clinical periodontitis. A dichotomous dependent variable indicated the presence of at least one symptom; an exploratory descriptive variable indicated two or more symptoms.

### 2.8. Data Collection Procedure

Eligible adults were informed about the study’s purpose and the voluntary nature of participation before receiving the questionnaire. Participants completed the form in a quiet waiting area or community setting without discussion with other respondents. Completion time averaged 8 to 10 min. The research team checked questionnaires only for missing pages or unintentionally skipped items; no attempt was made to influence responses. Each form was coded numerically to preserve anonymity.

### 2.9. Statistical Analysis

Data were analyzed using IBM SPSS Statistics version 25.0 (Armonk, NY, USA). Continuous variables were summarized as mean ± standard deviation; categorical variables were expressed as *n* and percentage. Normality of awareness scores was evaluated using histograms, skewness-kurtosis values, and the Kolmogorov–Smirnov test. Between-group comparisons for categorical variables were performed using Pearson chi-square tests. Mean awareness scores were compared using independent-samples *t* tests or one-way ANOVA with Tukey post hoc tests. Predictors in the multivariable logistic regression model were selected a priori based on periodontal relevance and previous literature: age group, sex, education level, smoking status, brushing frequency, interdental cleaning, and dental attendance pattern [11,12,13,14,15,16,17,18,19,24,25,26,27,32]. Variables associated with symptoms at *p* < 0.10 in univariable analysis were also considered. Adjusted odds ratios (ORs) and 95% confidence intervals (CIs) were reported. Statistical significance was set at *p* < 0.05.

## 3. Results

A total of 386 questionnaires were included in the final analysis. The mean age was 31.8 ± 9.6 years (range: 18–65 years), and 224 respondents (58.0%) were women. More than half of the sample had university-level education, and 24.4% were current smokers. Detailed demographic characteristics are presented in Table 1.

With regard to plaque-control behavior, 56.0% reported brushing twice daily and 38.6% used interdental cleaning aids. Only 28.5% reported routine dental check-ups every six months, whereas 42.5% sought dental care only when a complaint occurred (Table 2). 

The mean awareness score was 12.7 ± 3.8. Low awareness was observed in 22.0%, moderate awareness in 50.5%, and high awareness in 27.5%. Correct recognition that gingival bleeding is not normal was reported by 72.0%, whereas only 46.1% correctly identified smoking as increasing periodontal disease risk (Table 3).

Awareness scores differed significantly across behavior groups (Table 4). Participants using interdental cleaning aids had higher mean awareness scores than non-users (14.8 ± 3.2 vs. 11.4 ± 3.6, *p* < 0.001). Routine preventive dental attendance and higher education were also associated with higher awareness scores.

Gingival bleeding was the most commonly reported symptom (47.4%), followed by dentin hypersensitivity (33.9%), halitosis (29.8%), and food impaction (28.8%). The prevalence of at least one symptom was higher among non-users of interdental cleaning aids (71.7%) than among users (49.7%; *p* < 0.001) (Table 5).

Because education level and dental attendance were also associated with awareness, additional analyses were performed for the composite symptom outcome. At least one symptom was most frequent among participants with complaint-based dental attendance and among those with high-school-or-lower education (Table 6).

**Figure 1:**
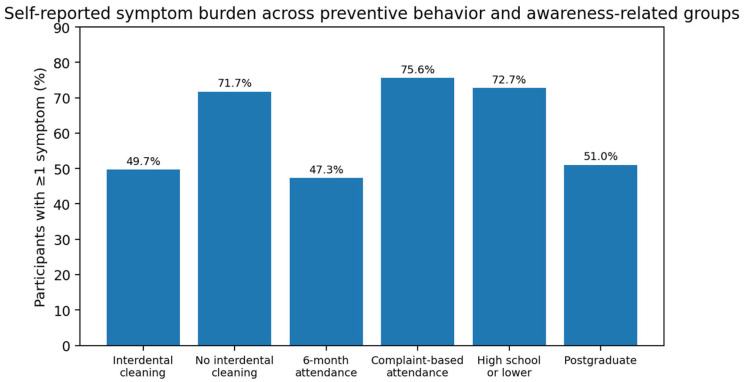
Prevalence of reporting at least one periodontal symptom across selected behavior and awareness-related groups.

Figure 1 summarizes the differences in self-reported symptom burden across selected preventive behavior and awareness-related groups.

In the multivariable logistic regression model, complaint-based dental attendance, current smoking, and lack of interdental cleaning remained significant independent correlates of self-reported symptom burden (Table 7). Brushing once daily or less showed an elevated odds ratio but borderline statistical significance after adjustment.

## 4. Discussion

This cross-sectional survey explored the intersection of oral-hygiene behavior, periodontal awareness, and self-reported symptom experience in adults. The overall pattern of results suggests that awareness and preventive behavior were associated in the same direction, but not perfectly. Participants with interdental cleaning habits and routine dental attendance had higher awareness scores (Table 4), whereas participants without interdental cleaning and those with complaint-based attendance reported more symptoms (Table 5 and Table 6).

The mean awareness score indicated moderate rather than robust awareness. Participants generally recognized obvious signs such as gingival bleeding, but showed weaker understanding of less visible or multifactorial elements such as smoking-related periodontal risk. This finding is consistent with previous work showing that self-reported periodontal knowledge and symptoms can be informative but should not be equated with clinical diagnosis [16,17,18,19,20,21,22,23].

The statement that individuals may respond to bleeding only when it becomes frequent is an interpretive explanation rather than a direct questionnaire item in the present study. It is supported indirectly by the moderate awareness score, the high prevalence of bleeding, and previous literature showing global periodontal burden and discordance between perceived and objective periodontal treatment need [24,37,38]. This clarification was added to avoid implying that frequency-based bleeding behavior was directly measured.

The association between interdental cleaning and awareness score deserves attention. Toothbrushing is common, but interdental cleaning may better discriminate between basic and more advanced self-care routines. In the present study, “lower symptom burden” refers specifically to a lower prevalence of reporting at least one symptom and lower prevalence of most individual symptom items among interdental cleaning users compared with non-users (Table 5). This does not imply a clinically verified reduction in periodontitis.

Dental attendance pattern was another major correlate of both awareness and symptom experience. Participants attending routine check-ups every six months had substantially higher awareness scores than those who sought care only for pain or treatment need (Table 4), and complaint-based attendance showed the highest prevalence of at least one self-reported symptom (Table 6). This finding is consistent with public-health literature indicating that regular professional contact may reinforce preventive adherence and risk perception [25,26,27,32,33].

Smoking was associated with greater symptom burden in the adjusted model, which aligns with the established role of tobacco as a major periodontal risk factor [12,13,14,15]. The finding that smoking-related periodontal knowledge was suboptimal also suggests that tobacco-related periodontal counseling should be integrated more explicitly into patient education strategies.

Educational attainment showed a positive relationship with awareness score, although its independent contribution to symptom reporting was attenuated in multivariable analysis. This pattern is compatible with social-determinants and common-risk-factor frameworks, whereby education may influence periodontal outcomes indirectly through health literacy, preventive orientation, access to information, and service utilization [27,28].

From a preventive periodontology perspective, the findings support a multi-component intervention model. Patient education should clarify that gingival bleeding is not normal, that periodontitis may progress with limited pain, and that interdental cleaning and preventive recall are central components of plaque-control behavior. Questionnaire-based screening may help identify high-risk behavior profiles, especially where full-mouth examinations are impractical [18,19,20,21,22,23].

This study has limitations. First, the cross-sectional design precludes causal inference. Second, all behavior and symptom variables were self-reported and therefore subject to recall bias and social desirability bias. Third, the composite symptom outcome was intentionally broad; halitosis, dentin hypersensitivity, and food impaction are not specific markers of periodontitis. Fourth, convenience sampling may overrepresent adults already connected to dental services. Future studies should combine questionnaire data with clinical periodontal examination and use longitudinal designs to determine temporal relationships.

## 5. Conclusions

1. In this cross-sectional survey, interdental cleaning and regular preventive dental attendance were associated with higher periodontal awareness scores.
2. Self-reported periodontal symptom burden was higher among current smokers, complaint-based dental attenders, and participants who did not use interdental cleaning aids.
3. Because the study was observational and questionnaire-based, the findings should be interpreted as associations and not as evidence that awareness or any specific behavior caused the observed symptom differences.
4. Questionnaire-based assessment may help identify knowledge gaps and preventive behavior profiles, but clinical periodontal examination remains necessary for diagnosis.

## Figures and Tables

**Table 1:** Demographic characteristics of the study population.

Characteristic	*n*	%
Sex: Women	224	58.0
Sex: Men	162	42.0
Age group 18–24 years	96	24.9
Age group 25–34 years	154	39.9
Age group 35–44 years	82	21.2
Age group ≥45 years	54	14.0
Education: High school or lower	121	31.3
Education: University	214	55.4
Education: Postgraduate	51	13.2
Current smoking	94	24.4
Previous professional scaling/periodontal treatment	138	35.8

**Table 2:** Oral hygiene behaviors and dental attendance patterns.

Behavior Variable	Category	*n*	%
Toothbrushing frequency	≤1 time/day	123	31.9
	2 times/day	216	56.0
	≥3 times/day	47	12.2
Brushing duration	<2 min	134	34.7
	2 min	177	45.9
	>2 min	75	19.4
Interdental cleaning aid use	Yes	149	38.6
	No	237	61.4
Mouthrinse use	Yes	138	35.8
	No	248	64.2
Dental attendance	Every 6 months	110	28.5
	Once yearly	112	29.0
	Only when complaint occurs	164	42.5

**Table 3:** Correct response frequency for periodontal awareness items.

Awareness Item	Correct (*n*)	Correct (%)
Dental plaque can cause gingival inflammation.	301	78.0
Bleeding during toothbrushing is not a normal finding.	278	72.0
Periodontitis can lead to tooth mobility and tooth loss.	252	65.3
Interdental cleaning is beneficial in addition to toothbrushing.	243	63.0
Gingival recession may indicate periodontal tissue loss.	221	57.3
Smoking increases periodontal disease risk.	178	46.1
Regular professional maintenance helps prevent periodontal deterioration.	247	64.0
Periodontal disease may progress with little pain.	216	56.0
Halitosis can be associated with gingival/periodontal problems.	233	60.4
Plaque control is important even in the absence of pain.	278	72.0

**Table 4:** Comparison of mean periodontal awareness score across selected variables.

Variable	Group	Mean Awareness Score ± SD	*p* Value
Education level	High school or lower	10.9 ± 3.5	<0.001
	University	13.3 ± 3.4	
	Postgraduate	15.0 ± 3.1	
Interdental cleaning	Yes	14.8 ± 3.2	<0.001
	No	11.4 ± 3.6	
Dental attendance	Every 6 months	15.1 ± 3.0	<0.001
	Once yearly	12.8 ± 3.4	
	Only when complaint occurs	11.3 ± 3.7	
Smoking status	Current smoker	11.5 ± 3.9	0.002
	Non-smoker/former smoker	13.1 ± 3.7	

**Table 5:** Distribution of self-reported periodontal symptoms according to interdental cleaning habit.

Self-Reported Symptom	Overall *n* (%)	Interdental Cleaning “Yes” *n* (%)	Interdental Cleaning “No” *n* (%)	*p* Value
Gingival bleeding	183 (47.4)	52 (34.9)	131 (55.3)	<0.001
Gingival swelling	91 (23.6)	24 (16.1)	67 (28.3)	0.007
Dentin hypersensitivity	131 (33.9)	40 (26.8)	91 (38.4)	0.022
Halitosis	115 (29.8)	32 (21.5)	83 (35.0)	0.006
Food impaction	111 (28.8)	31 (20.8)	80 (33.8)	0.008
Gingival recession	86 (22.3)	25 (16.8)	61 (25.7)	0.045
Tooth mobility	34 (8.8)	8 (5.4)	26 (11.0)	0.071
At least one symptom	244 (63.2)	74 (49.7)	170 (71.7)	<0.001

**Table 6:** Prevalence of at least one self-reported periodontal symptom according to education and dental attendance.

Variable	Group	At Least One Symptom *n*/*N* (%)	*p* Value
Education level	High school or lower	88/121 (72.7)	0.009
	University	130/214 (60.7)	
	Postgraduate	26/51 (51.0)	
Dental attendance	Every 6 months	52/110 (47.3)	<0.001
	Once yearly	68/112 (60.7)	
	Only when complaint occurs	124/164 (75.6)	

**Table 7:** Multivariable logistic regression analysis for presence of at least one self-reported periodontal symptom.

Predictor	Adjusted OR	95% CI	*p* Value
Complaint-based dental attendance vs. regular 6-month attendance	2.43	1.46–4.03	0.001
Once-yearly attendance vs. regular 6-month attendance	1.41	0.84–2.36	0.192
Current smoking	1.96	1.17–3.29	0.011
No interdental cleaning	2.08	1.31–3.30	0.002
Brushing ≤ 1 time/day	1.56	0.96–2.54	0.073
University/postgraduate education	0.78	0.49–1.26	0.313
Age ≥ 35 years	1.19	0.74–1.93	0.471
Female sex	0.93	0.59–1.48	0.765

## Data Availability

The data presented in this study are available upon request from the corresponding author in compliance with institutional ethical guidelines and data protection regulations.

## Appendix A

**Table A1:** Questionnaire structure and scoring.

Domain	Number of Items	Examples of Variables
Sociodemographic/ background	7	Age, sex, education, smoking status
Oral-hygiene behavior and dental attendance	12	Brushing frequency, duration, interdental cleaning, mouthrinse, dental visits
Periodontal awareness	10	Bleeding abnormality, plaque, interdental cleaning, smoking risk, recession, mobility
Symptoms and periodontal care history	13	Bleeding, swelling, hypersensitivity, halitosis, food impaction, recession, mobility, previous scaling/treatment

The administered questionnaire contained 42 items. Response formats were yes/no, multiple-choice, or true/false/“I do not know”. The awareness score was calculated from 10 true/false/“I do not know” items; correct responses were scored 2 and incorrect/“I do not know” responses were scored 0. Total score range was 0–20. Cronbach’s alpha for the awareness items was 0.81.
